# Head tracking extends local active control of broadband sound to higher frequencies

**DOI:** 10.1038/s41598-018-23531-y

**Published:** 2018-03-29

**Authors:** Stephen J. Elliott, Woomin Jung, Jordan Cheer

**Affiliations:** Institute of Sound and Vibration Research, University of Southampton, Southampton, SO17 1BJ United Kingdom

## Abstract

Local active sound control systems provide useful reductions in noise within a zone of quiet which only extends to about one tenth of an acoustic wavelength. If active control is required above a few hundred hertz, this generally limits the movement of a listener to unrealistically small changes in head position. We describe a local active sound control system using a fixed array of monitoring microphones, in which the pressures at the ear positions are estimated from these microphone signals using head position information from an optical head tracker. These signals are then actively controlled to give robust attenuation at the ear positions, even as the listener moves their head. Feedforward control provides selective attenuation of noise and broadband attenuation of around 20 dB is measured up to excitation frequencies of 1 kHz under favourable conditions, with head tracking achieved in a few seconds. The active control performance is thus comparable with that achieved with active headphones, but without the listener having anything attached to their head.

## Introduction

Although active sound control headphones, which can reduce noise levels for frequencies up to about 1 kHz, are now widely available, their use is not convenient in many applications. Examples are when the listener is driving, or when sound control is required over long periods of time, in which case headphones can become uncomfortable. Local active control of sound is also possible using loudspeakers and microphones remote from the ears, particularly if virtual sensing algorithms are used^1–7^. The zone of quiet around such a virtual microphone position, within which an attenuation of 10 dB is achieved, is limited to a diameter which is typically about 1/10 of an acoustic wavelength, however^8,9^. At frequencies above a few hundred hertz, normal changes in head position, of 5 cm or so, will thus move the ears out of this zone of quiet and effective sound control will be lost.

**Figure 1:**
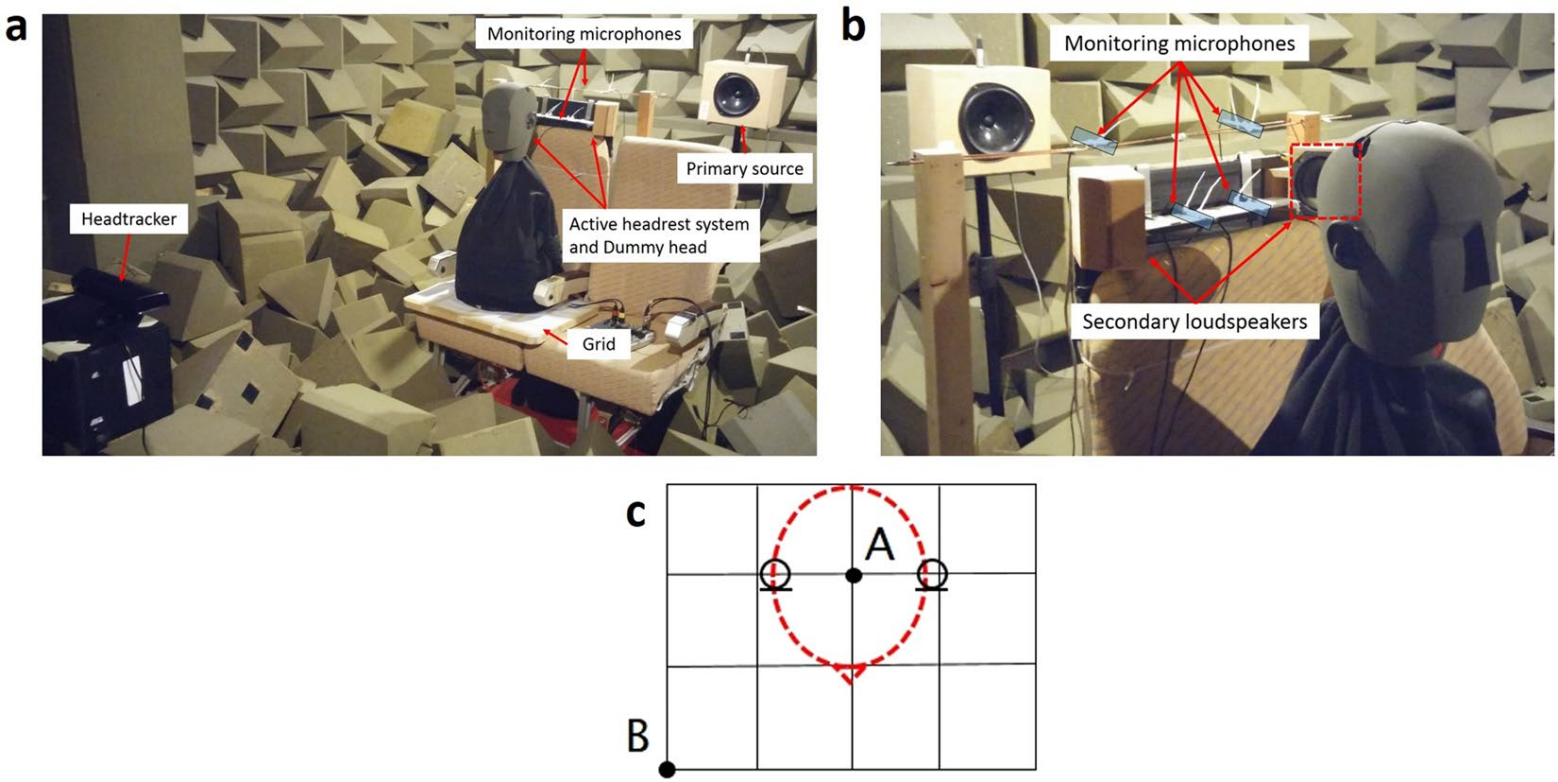
Physical arrangement used to demonstrate local active control at the ears of a listener while the head position is being tracked (**a**) and detail showing the position of the monitoring microphones and secondary loudspeakers (**b**), together with the grid, with a spacing of 5 cm, used to define the 20 measurement locations for the centre of the dummy head, including the nominal position, A, and the moved position, B, used in the following experiments (**c**).

In this paper, a system is described in which the listener’s head movements are tracked optically and this information is used by a local active control system to ensure that the broadband random sound at the listener’s ears is attenuated at frequencies up to about 1 kHz. A motivating application is the local active control of mid-frequency road noise in ears, from about 300 to 1,000 Hz, since it is known that global active control systems work well below 300 Hz^10^. Although the concept of using head tracking with local control was suggested some time ago^7^, and a similar system has recently been described for the active control of tonal noise^11^, the results presented here, for the active control of broadband noise, clearly demonstrate the range of frequencies over which such active control is possible. A headrest system is used for the demonstration^3^, with two secondary loudspeakers at the sides of the headrest and a static array of four monitoring microphones along the top of the headrest, as shown in Fig. 1. The remote microphone technique^7,12^ was used to estimate the pressures at the ears of the dummy head from those at the monitoring microphones. The responses from the loudspeakers to the microphones at the ear positions of a dummy head, which are required to implement this technique, were measured in an initial calibration phase when the dummy head was moved to 20 pre-defined positions on a grid. The positions of the four monitoring microphones were chosen from a larger number of potential monitoring microphone positions after a series of preliminary experiments, which showed that these positions gave the best performance for a number of different primary source locations. Several methods of selecting the positions of these microphones have recently been reported^13^. In the experiments here, two monitoring microphones were used lower down on the headrest and two higher up, as shown in Fig. 1(b), and these microphones were about 18 cm and 27 cm, respectively, away from the centre of the dummy head when it was in the nominal position, which is shown as position A on the grid in Fig. 1(c).

## Results

**Figure 2:**
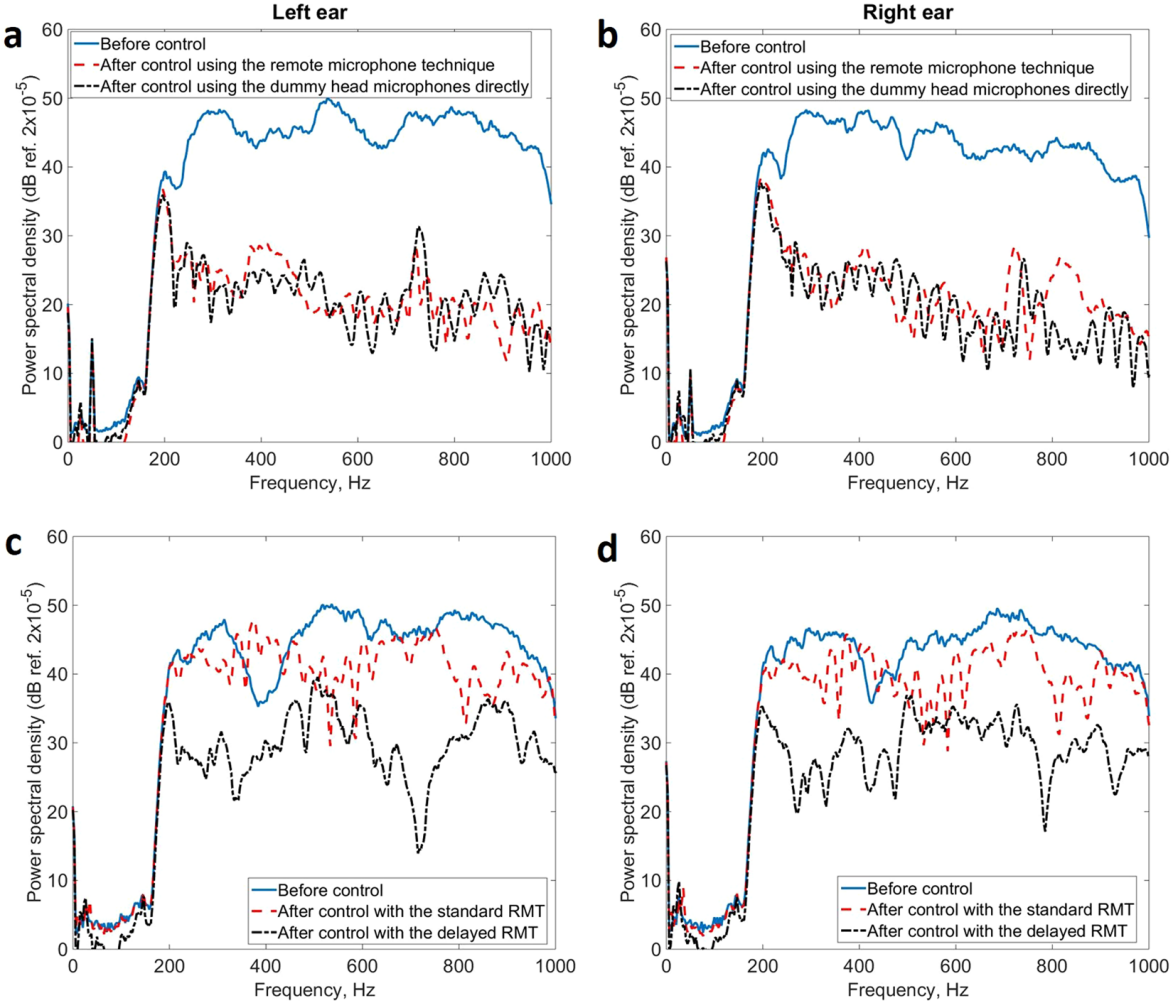
Power spectral density of the signal measured at the microphones in the left and right hand side of the dummy head when it was in the nominal position and the primary source was behind the headrest before control, solid line, after control using the monitoring microphones and observation filter to estimate the signal at the ear position using the remote microphone technique, dashed line, and, for reference, when the microphones in the dummy head themselves were used as the error signals in the control algorithm, dot-dashed line (**a**) and (**b**). The results are also shown when the primary source was positioned in front of the headrest in the two lower graphs, (**c**) and (**d**), which illustrate the effect of the standard remote microphone technique, RMT, and when it includes a modelling delay of 0.7 ms.

The loudspeaker acting as the primary source was initially located behind the headrest, as in Fig. 1, and driven by band-limited Gaussian white noise between 200 and 1,000 Hz. The power spectral density of the pressure signals from the microphones in the left and right-hand ear positions of the dummy head was then measured, as shown by the solid lines in Fig. 2(a) and (b). The dummy head was in the nominal position, A in Fig. 1, and this was identified by the head tracker before control and the appropriate set of observation filters and plant response filters was used in the control algorithm to attenuate the pressure at the two ears. As a benchmark for the best possible performance with this arrangement, control was also implemented using the signals from the microphones mounted in the dummy head directly as error signals, with the results also shown in Fig. 2(a) and (b). Reductions in pressure of about 20 dB are observed at the error microphones on both sides of the dummy head after active control, from about 300 Hz to about 1 kHz. Below 300 Hz, the small loudspeakers used as secondary sources are not very efficient and lower levels of control are achieved. The results obtained using the monitoring microphones and remote microphone technique, as shown by the dashed lines in Fig. 2(a) and (b), are almost as good as those obtained by directly minimising the signal from the error microphones in the dummy head, as shown by the dot-dashed lines in these figures. Tests were also performed with the loudspeaker acting as the primary source at other positions, including in front of the headrest, with the results shown in the lower two graphs in Fig. 2. It was important to include a suitable modelling delay in the observation filters for the front primary source locations, since otherwise the observation filters are non-causal and, as can be seen from the dashed lines in Fig. 2(c) and (d), the attenuation is limited when the standard remote microphone technique, with no modelling delays, is used.

**Figure 3:**
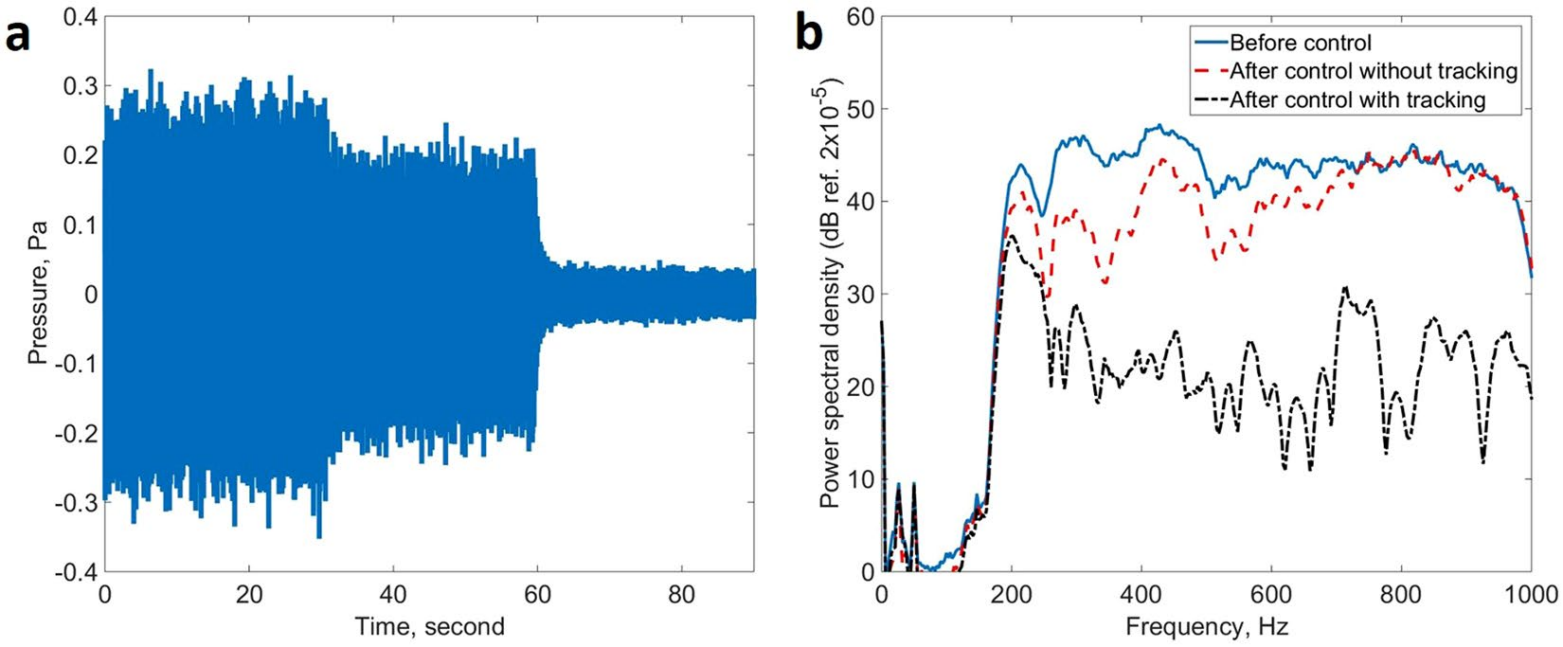
Time history of the pressure signal measured in the right-hand ear of the dummy head, (**a**) when in position B before control, up to 30 seconds, when control is implemented with the observation filter and plant responses appropriate for the nominal head position, from 30 to 60 seconds, and when the head tracker is enabled so that the correct head position is identified, after 60 seconds. The power spectral density of the signal at this microphone is also shown, (**b**) before control, after control but without head tracking and after control with the head tracker enabled.

The head tracking performance was then tested by moving the dummy head from the nominal position to other head positions. Figure 3 shows the time history of the signal from the right-hand ear of the dummy head after it had been moved to position B on the grid shown in Fig. 1, which is about 14 cm from the nominal head position, A. During the first interval shown in Fig. 3(a), up to 30 seconds, the active control system is switched off, so that the measured pressure is just due to the disturbance from the loudspeaker acting as the primary source behind the headrest. The control system is then switched on, from 30 to 60 seconds, but with the observation filter and plant responses appropriate to the head position still being in the nominal head position, A. The results shows some reduction in level, but this is not as great as when the head tracker was switched on, after 60 seconds. Figure 3 shows that once the head tracking system is enabled at 60 seconds, the correct head position is acquired and the observation filter and plant responses are updated within a second or so to give good control.

The spectrum of the pressure at the right-hand ear of the dummy head is shown in Fig. 3(b) under these three conditions. It can be seen that without head tracking, attenuations of about 10 dB are achieved up to only about 600 Hz, whereas with head tracking enabled, attenuations similar to those achieved with the head at the nominal position, in Fig. 2, are obtained. Experiments have also been performed moving the dummy head to other locations, with similar results to those shown in Fig. 3, except that at some head locations the disturbance signal is actually enhanced, before head tracking is implemented and the filters updated, especially at higher frequencies. If the head movement is too large, it is also possible for the control system to become unstable without head tracking.

**Figure 4:**
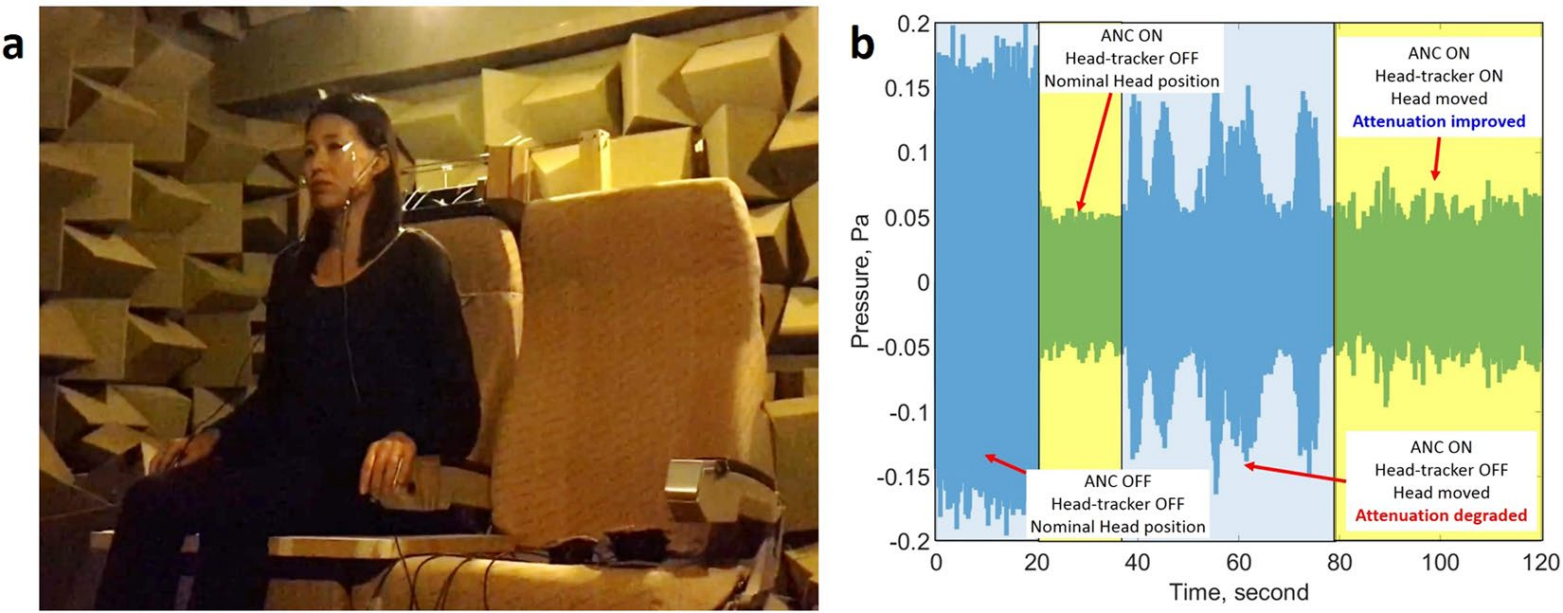
Active control with the integrated active headrest system with a human listener for reducing broadband random disturbance signals in real-time: (**a**) The test installation, in which the participant has two physical error microphones in their ears for evaluation purposes and (**b**) the measured signals at one of these error microphones during active control and head-tracking.

Further experiments have been conducted with a human listener moving their head within the area defined by the grid above, when they were wearing in-ear microphones to monitor the pressure at their ear positions. The results are shown in Fig. 4, with a video of these experiments available in the supplementary material.

## Discussion

The aim of this work was to investigate the use of head tracking to steer the positions at which local active sound control is obtained to be close to the ears of the user for broadband noise disturbances. Without head tracking, the zones of quiet are fixed, and the subjective impression of listening in the headrest is that of moving in and out of the zones of quiet, as the head is moved by 5 cm or so. With head tracking, the zones of quiet are moved with the ear locations as the head position is changed and good cancellation is maintained even during head motion. Control is achieved up to about 1 kHz, at which the theoretical size of the zone of quiet in a diffuse field, one tenth of a wavelength^8,9^, is about 3.4 cm. In the present arrangement, the spacing between the identified head positions is about 5 cm, which is slightly larger than the diffuse field zone of quiet but since these experiments are performed under anechoic condition, the zone of quiet is slightly larger^14^ and so good control is maintained at positions between the identified head positions.

The main objective of this paper is to demonstrate the capacity for head tracking to extend the frequency range of control for broadband noise, and so these initial experiments are deliberately rather idealised. There are a number of practical applications for such a local active system for broadband noise control and several issues may need to be addressed in these cases, which are not considered in detail here, since the solutions are likely to be rather application-specific. In particular, we note that:

- First, direct access has been assumed to the primary source in order to provide a reference signal. In many practical applications, such as the control of road noise in cars^10^, this is not realistic and the reference signals have to be obtained from carefully located sensors placed on the vehicle. The type and position of the sensors is still a matter of active investigation in this and other applications.
- Secondly, it has been assumed in the experiments reported here that only a single broadband primary source is present, whereas in many practical applications there will be several, partially correlated, primary sources contributing to the disturbance signals. This kind of excitation would generate a more diffuse primary sound field than was experienced in the experiments described here, which will influence the accuracy with which the observation filter can estimate the signals from the error microphones. This has, to some extent, been taken into account here by selecting the position of the measurement monitoring microphones based on their performance when a number of different primary source locations are considered, but further work on this is clearly required when considering a specific application.
- Finally, the head tracking has been achieved here using a commercial system and we have only tracked the translational motion of the head. In practice the head will probably have rotational as well as translational motion, which will need to be tracked, and depending on the application, the speed of the head tracking system may be important in following rapid head movement.

One of the greatest barriers to the practical implementation of active noise control in vehicles, however, is cost. It is therefore significant that a number of automotive manufacturers are beginning to install head tracking devices in their vehicles to provide additional functionality, such as monitoring the alertness of drivers for example.

## Methods

**Figure 5:**
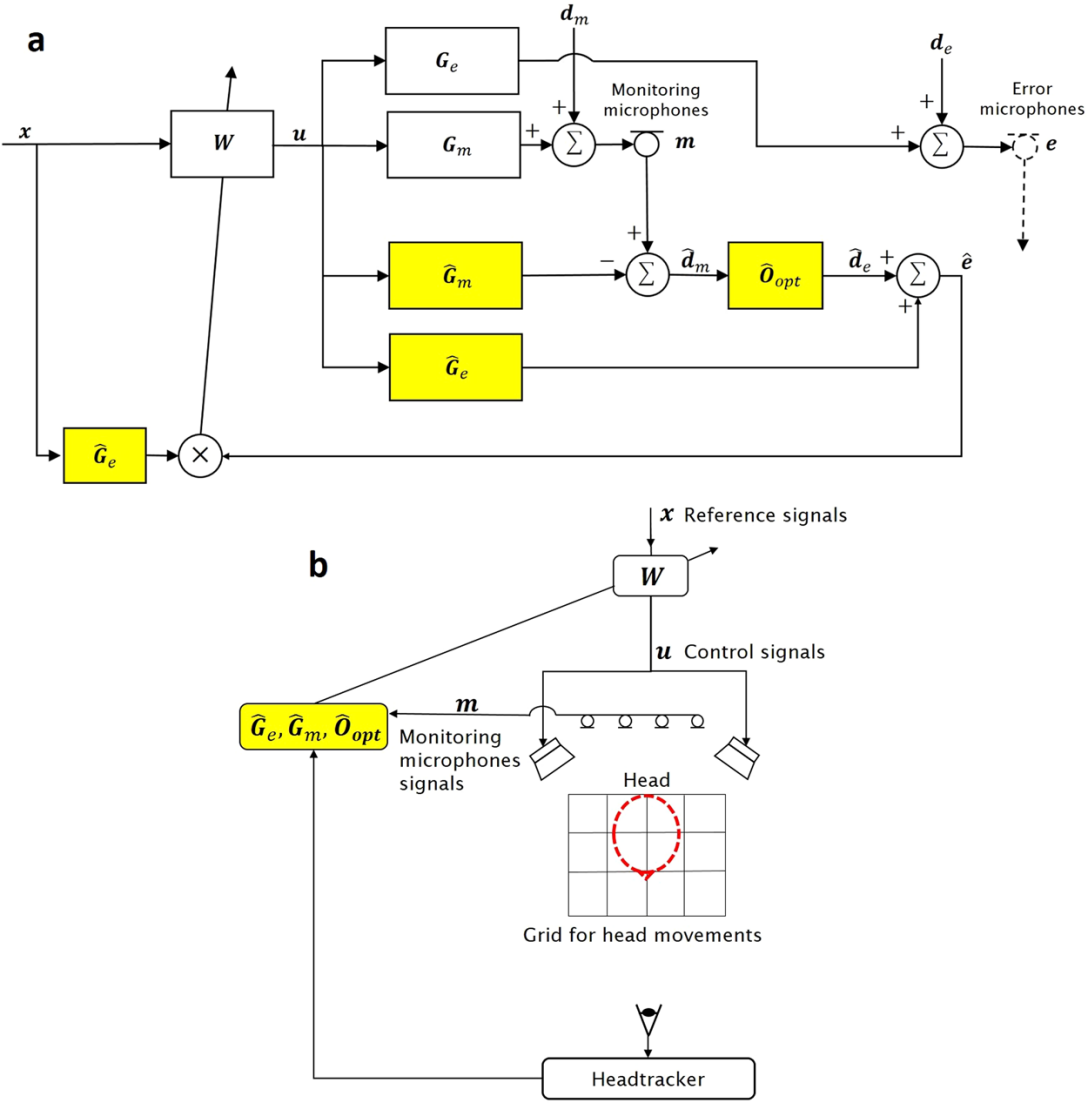
The block diagram of the adaptive control algorithm that uses the remote microphone technique to estimate the signals at the virtual error microphones from those at the fixed monitoring microphones, (**a**) and the arrangement used to implement the control system with a headtracker to schedule the transfer responses shown highlighted in the block diagram (**b**). The shaded blocks in both diagrams are the responses that are scheduled on the head position, as monitored by the headtracker.

The block diagram of the adaptive control algorithm used to control the broadband random pressures at the ears of the dummy head is shown in Fig. 5. The signals are generally vectors and the transfer responses are generally matrices in this diagram, in which ***x*** denotes the vector of reference signals related to the primary sources, which are assumed to be observed directly in these experiments. These reference signals are filtered by the FIR control filters in the matrix ***W***, driven by the reference signals, ***x***, to give the vector of control signals driving the two secondary loudspeakers, ***u***. The observed signals at the monitoring microphone positions are produced by the summation of the random disturbance signals at these positions, ***d***_*m*_, and the control signals filtered by the plant response **G**_*m*_. The unobserved signals at the virtual error sensors, ***e***, are given by the sum of the random disturbances at these positions, ***d***_***e***_, and the control signals filtered by the plant responses **G**_***e***_. The microphones in the two ears of the dummy head were used as error sensors during the initial training of the control system. The signals at the virtual error sensors are then estimated during control using the remote microphone technique^7,12^. The disturbance signals at the monitoring microphone are first estimated by subtracting an estimate of the contribution from the secondary source signals, obtained by filtering the control signals with Ĝ*m*, which is an estimate of **G**_*m*_. These estimates of the disturbances at the monitoring microphones, ${\hat{{d}}}_{m}$, are then filtered by an observation filter to estimate the disturbance signals at the virtual microphone locations. Finally, the error signals at the virtual microphone locations are calculated by adding an estimate of the contribution from the secondary sources at this point, using, Ĝ*e*, which is an estimate of **G**_*e*_.

The observation filter that optimally estimates the disturbance signals at the virtual error microphones from those at the physical monitoring microphones can be calculated using a least-squares formulation^11,14^. It is given by a *N*_*m*_ by *JN*_*m*_ matrix of filter coefficients, where *N*_*m*_ is the number of monitoring microphones, *N*_*e*_ is the number of virtual error microphones, and *J* is the order of each individual FIR observation filter, which is equal to

1 $${{\bf{O}}}_{{\rm{opt}}}={({\{{{\boldsymbol{R}}}_{{mm}}{+}{\beta }{\bf{I}}\}}^{-1}\{{{\boldsymbol{R}}}_{{me}}\})}^{{\rm{T}}},$$

where ***R***_*mm*_ is the *JN*_*m*_ by *JN*_*m*_ matix of auto- and cross-correlations between the delayed disturbance signals at the monitoring microphones and ***R***_*me*_ is the *JN*_*m*_ by *N*_*e*_ matrix of cross-correlations between the delayed disturbance signals at the monitoring microphones and the disturbance signals at the error microphones. The regularisation parameter, *β*, is chosen to give a reasonable trade-off between the accuracy with which the disturbance signals are estimated at the virtual microphone positions and the robustness of this estimate to small changes in these positions and to the locations of the primary source. The 8 individual observation filters in the matrix **O**_opt_ that were used in the arrangement here each had 128 coefficients and were identified for each of the 20 head locations on the grid. It should again be emphasised that in contrast to the remote active control of tonal noise in^11^, where only the in phase and quadrature components of a single frequency reference signal have to be adapted to achieve control, the real-time control of broadband random noise requires digital filters for the controller, plant models and observation filter, in order to achieve control over a range of frequencies.

The filtered-reference LMS algorithm^15,16^ used to update the control signal filters is

2 $${\boldsymbol{w}}(n+\mathrm{1)=}{\boldsymbol{w}}(n)-\alpha {\hat{{\boldsymbol{R}}}}^{T}({n})\hat{{\boldsymbol{e}}}({n}),$$

where ***w***(*n*) is the vector of controller coefficients at the *n*-th sample, ${\hat{{\bf{R}}}}^{T}(n)$ is the transpose of the matrix of reference signals filtered by the responses in Ĝ_*e*_, and ***ê***(n) is the vector of estimated error signals at the virtual error microphones. The control system used 128 coefficients for each of the four control filters and was implemented on a dSPACE system at a sampling rate of 3 kHz, with anti-aliasing and reconstruction filters having a cut-off frequency of 1 kHz. A Microsoft Kinect (Kinect 1.0 for Windows), was used for head tracking, positioned about 1.5 m away from the dummy head. The information from the Kinect was decoded in real-time using plug-in software implemented in MaxMSP and this was passed to the dSPACE controller, which already stored the various plant responses and pre-calculated observation filters for the 20 head positions.

Using a frequency domain analysis, a sufficient condition for the stability of this adaptive algorithm can be derived^17,18^, as

3 $${\rm{Re}}({\rm{eig}}[{\hat{{\bf{G}}}}_{e}^{H}{\hat{{\bf{G}}}}_{e}+{\hat{{\bf{G}}}}_{e}^{H}\,{\hat{{\bf{O}}}}_{{\rm{opt}}}({{\bf{G}}}_{m}-{\hat{{\bf{G}}}}_{m})])\mathrm{ > 0}.$$

As the position of the listener’s head changes, then clearly the observation filter and the responses from the secondary sources to the virtual positions of the error microphones will also change. It is also found, however, that the responses from the secondary sources to the fixed monitoring microphones, **G**_*m*_, also changes somewhat, due to the physical presence of the head, and that these changes can compromise the accuracy of the estimated error signals, and the stability of the system. The potential effect of a difference between **G**_*m*_ and **Ĝ**_*m*_ on the stability of the system can be seen from equation (3) to be particularly significant if the observation filter has a large response at some frequencies. This emphasises the need for regularisation in the design of the observation filters. Both **Ĝ**_*m*_ and **Ĝ**_*e*_ were modelled here as 64 coefficient FIR filters, at each of the 20 dummy head positions and both were scheduled on the measured head position.

In a series of experiments conducted prior to control, all of these three responses, **Ĝ**_*m*_, **Ĝ**_*e*_ and **Ô**_opt_, are identified as FIR filters from measurements made with the dummy head in each of the 20 separate positions on the grid shown in Fig. 1 and these are then stored in the control system. During real-time control, the head tracker is used to measure the head position and the closest pre-calculated head position is identified, as shown in Fig. 5(b). The responses associated with this head position are then used in the control algorithm shown in Fig. 5(a).

In operation, both an observation filter and the plant responses are selected based on the tracked head position, although it would also be possible to interpolate a larger number of these from the more limited number of measured responses. The feedforward control approach provides selective attenuation, only for sounds that are coherent with the reference signals. Although the usual causality issues associated with the time-advance of the reference signals are still present, causality problems associated with the estimation of the remote microphone signals are avoided by using the observation filter to calculate a delayed version of these signals^13,19^.

The convergence time of the adaptive algorithm in equation (2) depends on the overall delay in the plant response, **Ĝ**_*e*_^17^, which includes both the modelling delay and also the acoustic and electrical delays of the system. The modelling delay used for the observation filter in these experiments was 0.7 ms, which was not large, compared with the delay due to the acoustic propagation between the loudspeaker and microphone and that due to the anti-aliasing and reconstruction filters in this case, which was about 1.3 ms in total. So although, in principle, the modelling delay will increase the convergence time of the adaptive control algorithm, this increase is small in the experiments reported here, since the overall delay is not dominated by the modelling delay and the disturbances is also stationary. The control filter thus only had to adapt when there was a change of head position, in which case the time taken for the head tracker to acquire a new position was significantly greater than the adaptation time of the filter. There may be some applications, with small acoustic delays and nonstationary disturbances, in which this modelling delay does become important in determining the convergence time and so limits the control performance.

All methods were carried out in accordance with the relevant guidelines and regulations of the University of Southampton and all experimental protocols were approved by the University of Southampton. Informed consent was obtained from the subject participated in the experiment for Fig. 4.

### Data Availability

The datasets generated and/or analyzed during the current study are available from the corresponding author on reasonable request.

## Electronic supplementary material

Supplementary video
